# Antioxidant and Anti-Inflammatory Activities of Essential Oils: A Short Review

**DOI:** 10.3390/molecules15129252

**Published:** 2010-12-15

**Authors:** Maria Graça Miguel

**Affiliations:** Faculdade de Ciências e Tecnologia, Universidade do Algarve, IBB, Centro de Biotecnologia Vegetal, Ed. 8, Campus de Gambelas, 8005-139 Faro, Portugal; E-Mail: mgmiguel@ualg.pt

**Keywords:** essential oils, antioxidant, anti-inflammatory

## Abstract

Essential oils are complex mixtures isolated from aromatic plants which may possess antioxidant and anti-inflammatory activities of interest in thye food and cosmetic industries as well as in the human health field. In this work, a review was done on the most recent publications concerning their antioxidant and anti-inflammatory activities. At the same time a survey of the methods generally used for the evaluation of antioxidant activity and some of the mechanisms involved in the anti-inflammatory activities of essential oils are also reported.

## 1. Introduction

An essential oil is defined internationally as the product obtained by hydrodistillation, steam distillation or dry distillation or by a suitable mechanical process without heating (for *Citrus* fruits) of a plant or some parts of it [1]. They are aromatic oily liquids, volatile, characterized by a strong odour, rarely coloured, and generally with a lower density than that of water. They can be synthesized by all plant organs (flowers, buds, seeds, leaves, twigs, bark, herbs, wood, fruits and root) and therefore extracted from these parts, where they are stored in secretory cells, cavities, canals, epidermic cells or glandular trichomes [2,3]. Essential oils only represent a small fraction of plant’s composition; nevertheless they confer the characteristics by which aromatic plants are used in the food, cosmetic and pharmaceutical industries [4].

The proportions of the components present in essential oils vary greatly. Major components can constitute up to 85% of the essential oils, while the remaining components can be present in only trace amounts [5]. The aroma of each oil results from the combination of the aromas of all components, and even minor oil constituents may have major organoleptic roles to play [6].

In addition to the extraction techniques reported above there are other ones that may be used for extracting the volatile fraction, nevertheless this cannot be called an “essential oil” in those cases. Such techniques include: vacuum distillation, solvent extraction combined off-line with distillation, simultaneous distillation-extraction (SDE), supercritical fluid extraction (SFE), and microwave-assisted extraction and hydrodistillation (MAE and MA-HD), static (S-HS), dynamic (D-HS) and high concentration capacity headspace (HCC-HS) sampling [1]. These authors in a synthetic way explain how all of these techniques operate.

Essential oils have a complex composition, containing from a dozen to several hundred components. The great majority of components identified in essential oils includes terpenes (oxygenated or not), with monoterpenes and sesquiterpenes prevailing. Nevertheless, allyl- and propenylphenols (phenylpropanoids) are also important components of some essential oils [7].

Capillary gas chromatography is the technique of choice for the analysis of essential oils due to the volatility and polarity of essential oil components, combining two different-polarity stationary phases. Identification of oil components is generally performed by chromatographic data (Kováts indices, linear retention indices, relative retention time, retention time locking) and/or by spectral data, mainly by mass spectrometry (GC-MS), as well as other techniques reported in a recent review articles [1].

The complexity of essential oils makes the quantification of their components arduous. According to the review article [1] there are at least four widely used approaches: relative percentage abundance, internal standard normalized percentage abundance, “absolute” or true quantification of one or more components using internal and/or external standards, and quantification by a validated method. A set of applications for each approach is given [8].

Biogenetically, terpenoids and phenylpropanoids have different primary metabolic precursors and are generated through different biosynthetic routes. The pathways involved in terpenoids are mevalonate and mevalonate-independent (deoxyxylulose phosphate) pathway, whereas phenylpropanoids originate through the shikimate pathway [9,10]. Some authors have reviewed the biosynthetic pathways of terpenoids and phenylpropanoids, respectively, the enzymes and enzyme mechanisms involved, and information about genes encoding for these enzymes [9,10].

Genetic engineering of metabolic pathways has given some promising results for improving the production of volatiles. For that purpose, bacteria, yeasts and plants have been genetically altered either for the production of terpenoids or shikimic acid-derived volatiles. In a recent review article it several results of diverse authors concerning the production of volatile metabolites by transgenic microorganisms and genetically engineered plants were compiled [11]. Some authors have concluded that this type of approach could be used successfully to generate noticeable levels of terpenoids. Nevertheless, engineering of some classes of this group of compounds is quite difficult owing to the pool of terpenoid precursors may not be sufficient for the production of substantial quantities of the desired compound [12].

In Nature, essential oils play an important role in the attraction of insects to promote the dispersion of pollens and seeds or to repel other ones. In addition, essential oils may also act as antibacterials, antivirals, antifungals, insecticides, herbicides, or have feeding deterrent effects against herbivores by reducing their appetite for such plants. Essential oils have also an important role in allelopathic communication between plants [3,13]. The detection of some of these biological properties needed for the survival of plants has also been the base for searching similar properties for the combat of several microorganisms responsible for some infectious diseases in humans and animals. This search intends to respond to the increasing resistance of pathogenic microbes to antibiotics.

Reichling *et al.* (2009) [14] have compiled the most important results about antibacterial and antiviral properties of essential oils published in the last decade. In this review, the essential oils against bacteria from the respiratory tract, anti-*Helicobacter pylori*, anti-*Mycoplasma pneumoniae*; essential oils against DNA virus: HSV1 (herpes simplex virus), HSV-2, NDV (Newcastle disease); or RNA virus: SARS-Cov (severe acute respiratory syndrome-associated coronavirus), and Junin virus were overviewed. In addition, essential oils have also revealed to be effective on the inhibition of growth and reduction in numbers of the more serious foodborne pathogens such as *Salmonella* spp., *E. coli* O157:H7 and *Listeria monocytogenes* [2].

The antioxidant activity of essential oils is another biological property of great interest because they may preserve foods from the toxic effects of oxidants [15]. Moreover, essential oils being also able of scavenging free radicals may play an important role in some disease prevention such as brain dysfunction, cancer, heart disease and immune system decline. Increasing evidence has suggested that these diseases may result from cellular damage caused by free radicals [16,17].

If essential oils are able to scavenge some free radicals, they can also act as anti-inflammatory agents, because one of the inflammatory responses is the oxidative burst that occurs in diverse cells (monocytes, neutrophils, eosinophils, and macrophages). Phagocytosis of bacteria, which occurs during inflammation, is accompanied by a dramatic increase in oxygen consumption resulting in the formation of superoxide anion radical (O_2_^•-^) which is quickly converted to hydrogen peroxide (H_2_O_2_), spontaneously, or by the enzyme superoxide dismutase. Hydrogen peroxide can then also be reduced by transition metal ions generating the hydroxyl radical (HO^°^), one of the strongest oxidizing agents that can rapidly react with polyunsaturated fatty acids, resulting the production of peroxyl radicals (ROO^•^). Peroxide hydrogen can also oxidise halide ions (Cl^-^) to hypochlorous acid (HOCl), which is a strong oxidant that can react with amines producing chloramines, some of them being very toxic [18,19,20,21,22]. These radicals are commonly known as ROS (reactive oxygen species). Nevertheless and during an inflammatory process, there is also the generation of other free radicals named RNS (reactive nitrogen species). Nitric oxide (^•^NO) and peroxynitrite anion (ONOO^-^) are two examples of this sort of radicals. Nitric oxide is produced in large quantities by the inducible nitric oxide synthases (iNOS) in activated macrophages and neutrophils during defence and immunological reactions. However this reactive species can also exerts its toxicity by generating the peroxynitrite anion after reacting with the superoxide anion radical [22,23,24,25].

Peroxynitrite is formed by phagocytes for killing invading microorganisms, nevertheless if in high concentrations can oxidise several host biomolecules by nitration with the consequent cellular damages being responsible for diverse diseases [26].

ROS and RNS are generated in phagocytes in order to neutralise the invading organisms, having therefore an important role in the host defence mechanism. Nevertheless, their overproduction may be responsible for damage at inflammatory sites. In addition these reactive species play important roles in inflammation by being trigger elements or by being signalling messenger molecules. ROS and RNS act as modulators of protein and lipid kinases and phosphatases, membrane receptors, ion channels, and transcription factors, including nuclear factor-κB (NF-κB), which regulate the expression of key cytokines [22]. This short review reports the potential of essential oils as antioxidants and anti-inflammatory agents, as well as the *in vitro* mechanisms involved in such properties.

## 2. How Does an Antioxidant Act?

Antioxidants may act as physical barriers to prevent ROS generation or ROS access to important biological sites (UV filters, cell membranes); chemical traps/sinks that “absorb” energy and electrons, quenching ROS (carotenoids, anthocyanidins); catalytic systems that neutralize or divert ROS [antioxidant enzymes SOD (superoxide dismutase), catalase, and glutathione peroxidase]; binding/inactivation of metal ions to prevent generation of ROS (ferritin, ceruloplasmin, catechins); and chain-breaking antioxidants which scavenge and destroy ROS (ascorbic acid, tocopherols, uric acid, glutathione, flavonoids) [27]. Therefore, and based on their mode of action, the antioxidants can be classified as primary, secondary or co-antioxidants. Primary antioxidants are able to donate a hydrogen atom rapidly to a lipid radical, forming a new radical, which is more stable. Secondary anti-oxidants react with the initiating radicals (or inhibit the initiating enzymes), or reduce the oxygen level (without generating reactive radical species). Therefore, these secondary antioxidants can retard the rate of radical initiation reaction by elimination of initiators. This can be performed by deactivating high energy species (singlet oxygen); absorbing UV light; scavenging of oxygen; chelating metal that catalyses free radical reaction, or inhibiting enzymes, such as peroxidases, NADPH oxidase, xanthine oxidase, among other oxidative enzymes [28].

Direct and indirect methods have been applied to determine the chain-breaking antioxidant activity of natural products. Direct methods are based on studying the effect of a tested product (e.g. food) containing antioxidants on the oxidative degradation of a testing system. The substrate of oxidation may be individual lipids, lipid mixtures (oils), proteins, DNA, blood plasma, LDL, and biological membranes. Homogenous lipids or microheterogeneous systems (micelles and liposomes) can be used depending on the solubility of samples. The indirect method studies the ability of antioxidant to scavenge some free radicals, which is not associated with the real oxidative degradation [29].

In direct methods, they can be based on the kinetic of lipid peroxidation or on the kinetic of non-chain process (direct competition methods). In the first case, two modes of lipid peroxidation can be used for testing the antioxidant activity: one of them is the autoxidation mode when the process is progressing spontaneously, with self-acceleration due to the accumulation of LOOH; the other mode is based on the use of the kinetic model of the controlled chain reaction. The utilization of thermo-labile azo-compounds [water-soluble 2,2’-azobis(2-amidinopropane) dihydrochloride (AAPH) and lipid-soluble 2,2’-azobis(2,4-dimethylvaleronitrile) (AMVN)] that decompose and produce active free radicals at moderate temperatures at any desired rate, which can be easily altered and controlled are two good examples greatly used [29]. When these thermo-labile azo-compounds are not used, and for accelerating lipid oxidation, increased partial oxygen pressure and temperature; addition of transition metal catalysts; exposure to light; and variable shaking and free radical sources may be used [5].

There are two very popular methods for monitoring lipid peroxidation: determination of conjugated dienes (absorbance at 234 nm) and the TBARS (thiobarbituric acid reactive substances) assay. This method measures the malonaldehyde formed after lipid hydroperoxide decomposition, which forms a pink chromophore with thiobarbituric acid (TBA). However, TBARS is representative of a rather late stage of lipid oxidation, when the oxidation substrate has undergone substantial transformation [29]. There are other methods, including the determination of peroxide value, iodine value, chromatographic measurement of volatile compounds, formic acid measurement [the Rancimat method in an automated test that measures the conductivity of low molecular weight fatty acids (formic acid) produced during the auto-oxidation of lipids at 100 ºC or above] [5].

In the direct competition methods, natural antioxidants compete for the peroxyl radical with a reference free radical scavenger. Examples of reference free radicals scavengers include the fluorescent natural protein *R*-phycoerithrin and the natural crocin, or fluorescein, which compete with the antioxidant sample for peroxyl radicals formed. These radicals may be produced through the addition of AAPH or AMVA. This is the principle of the so-called ORAC (oxygen radical absorbance capacity) protocol. Another example is the bleaching of β-carotene during the autoxidation of linoleic acid or in a controlled chain reaction by using a free radical initiator: AAPH [29].

Examples of free radicals which is not associated with the real oxidative degradation (indirect method) are 2,2’-azinobis(3-ethylbenzothiazoline-6-sulfonate) (ABTS) or 2,2-diphenyl-1-picrylhydrazyl (DPPH), both with different colour according to the redox status. Another example of indirect method is based on the reduction of Fe^3+^ to Fe^2+^, in the presence of 2,4,6-trypyridyl-*s*-triazine. This method is known as FRAP (ferric reducing antioxidant power). There are also methods based on the chemiluminescence of luminal in the presence of free radicals, which can be produced through AAPH [29].

In addition to the methods to determine the chain-breaking antioxidant activity, there are also the remaining methods for evaluating the capacity for scavenging other free radicals such as superoxide, hydroxyl, nitric oxide, peroxynitrite or for chelating metals (secondary antioxidants). Many methods have been developed and compiled in recent review articles, being also in some of them reported the advantages and disadvantages of those methods [27,30,31,32,33,34,35,36,37,38,39,40,41]. The antioxidant ability of the essential oils has also been evaluated using several methods. A recent review discusses the diversity of tests used by different authors for evaluating the antioxidant activity of essential oils [5].

## 3. Essential Oils as Antioxidants

### 3.1. Assays associated with lipid peroxidation

Lipid oxidation is a complex reaction that can be generated via three different pathways: 1. Non-enzymatic free radical-mediated chain reactions; 2. Non-enzymatic, non-radical photo-oxidations; 3. Enzymatic reactions [5].

The first pathway leads to initiation of rapidly progressing, destructive chain reactions, generating hydroperoxides and volatile compounds, generally through a three-phase process: initiation, propagation and termination. 

The initiation phase involves homolytic breakdown of hydrogen in the *α-*position relative to the fatty acid chain double bond, leading to the formation of an allyl radical. These species are highly unstable, short-lived intermediates that stabilize themselves by abstracting hydrogen from another chemical species or rapidly react with oxygen to form a peroxyl radical (propagation phase). In the propagation phase, peroxyl radicals formed can further oxidise the lipid, producing hydroperoxides. These are stabilised via double-bond rearrangement (electron deslocalization), originating conjugated dienes and trienes. These intermediates decompose, originating alcohols, aldehydes, alkyl formates, ketones, hydrocarbons, alkoxyl radicals and formic acid. All of these compounds are considered secondary products of lipid oxidation [5].

In assessing lipid peroxidation, several lipid substrates can be used. *i.e.* oils and fats, linoleic acid, fatty acid methyl esters and low-density lipoproteins (LDLs). The antioxidant activity in such systems can be detected by measuring the substrate and the oxidant consumption, and the intermediates or the final products formation [5].

Several tests are available for determining the primary and secondary products of lipid oxidation. From the most recent works published in scientific journals about the antioxidant activity of essential oils, it is possible to register such diversity on the evaluation of the antixodant activity of essential oils.

#### 3.1.1. Peroxidation level evaluation using the ferric thiocyanate

During linoleic acid oxidation, peroxides are formed (primary products of oxidation), which oxidise Fe^2+^ to Fe^3+^. The latter ions form a complex with thiocyanate, and this complex has a maximum absorbance at 500 nm. Therefore, high absorbance indicates high linoleic-acid oxidation. Using this method, some authors [42] found that *Lavandula angustifolia* Mill. oil from Australia was significantly more effective against lipid peroxidation than any of the other oils studied. Such oil was predominantly constituted by linalool and lynalyl acetate.

#### 3.1.2. Conjugated diene assay

The antioxidant effect of test substances can be evaluated by monitoring the conjugated diene formation at the early stage of lipid peroxidation. The hydroperoxides formed from methyl linoleate by oxidation at 40 ºC was measured by [43] over time, spectrophotometrically, at a wavelength of 234 nm (for conjugated diene absorption). The oils of *Thymus vulgaris* L. (thyme), [*Eugenia caryophyllus* (C. Spreng) Bull et Hare] (clove), and *Ocimum basilicum* L. (basil) had appreciable antioxidant activities, comparable to that of α-tocopherol, the reference chosen by the authors [43]. In the thyme oil *p*-cymene and thymol predominated; in the clove oil, eugenol and β-caryophyllene dominated; and in basil oil, linalool, isoanethole and eugenol constituted the major components. All of these components present in different relative percentages in the oils had similar ability for preventing lipidic peroxidation.

#### 3.1.3. β-Carotene bleaching test

The *β*-carotene bleaching method (coupled oxidation of *β*-carotene and linoleic acid) estimates the relative ability of antioxidant compounds in plant extracts to scavenge the radical of linoleic acid peroxide that oxidizes *β*-carotene in the emulsion phase. *β*-carotene in the absence of the antixodant undergoes a rapid decolourization since the free linoleic acid radical attacks the *β*-carotene, which loses the double bonds and, consequently, its orange colour.

From the seven Himalayan Lauraceae species, the essential oils of *Dodecadenia grandiflora* Nees, *Lindera pulcherrima* (Nees) Benth. ex Hook. f. and *Persea gamblei* (King ex Hook. f.) Kosterm were able to inhibit linoleic acid oxidation. Sesquiterpenoids dominated the oils. Furanodiene and germacrene D predominated in the oils of *Dodecadenia grandiflora*, whereas furanodienone and curzerenone constituted the major components of the *Lindera pulcherrima* oil [44]. The oils of *Persea gamblei* were constituted by β-caryophyllene, γ-gurjunene and β-cubenene. Such activities were independent on the presence of phenolic compounds in the essential oils.

Mighri *et al.* (2010) [45] studied the antioxidant activity of the essential oils of *Artemisia herba-alba* Asso. cultivated in Southern Tunisia. Diverse methods were used, and one of them was the β-bleaching test. They found four oil types: β-thujone, α-thujone, thujones (α + β), and 1,8-cineole/camphor/thujones (α + β). All of these oils exhibited weak antioxidant abilities for preventing the linoleic acid oxidation. The β-thujone-rich oil showed the best inhibition percentage (12.5%), nevertheless much lower than BHA (89.2%). The attributed such results to the absence of non-phenolic compounds [45].

The essential oils of different parts of *Myrtus communis* var. *italica* L. leaf, stem and flower were chemically evaluated. α-Pinene and 1,8-cineole dominated the leaf oil; 1,8-cineole, α-pinene, *trans*-caryophyllene and linalool constituted the major components of the stem oil, whereas in the flower oil, along with these 4 components, there was also α-terpineol and eugenol. Leaf and flower oils had the best antixidant activities, nevertheless inferior to those of BHT and BHA. The authors [46] attributed such weak activities to the low level of phenolic compounds (eugenol in the flowers) or even their absence (stem and leaf), because the extracts also studied in the present work presented higher activities. Nevertheless it is worthy to refer the similar activities found in leaf and flower oils, in spite of the absence or phenols in leaf oils.

The antioxidant activity of *Hymenocrater longiflorus* Benth. from Iran was evaluated [47]. The main components of the oils included α-pinene, 1,8-cineole, β-eudesmol, spathulenol, hedycaryol, δ-cadinene, among other components, nevertheless predominating the oxygenated sesquiterpenids (47.4%). The essential oils were able to inhibit the bleaching of β-carotene. The precentage of inhibition was even close (66.4%) to those found for the non-polar-sub-fraction (chloroformic) (69.1%), which presented the best activity. However, the authors did not explain these results which are contradictory to those obtained when the antioxidant activity was measured through other method, in which the polar fraction rich in phenolic compouds had the greatest activity [47].

The antioxidant activities of other oils isolated from diverse Iranian plants were also assayed [48,49]. In both cases the activities were weak. The percentage inhibition of *Salvia eremophila* Boiss. oil was of about one third of that of BHT, in contrast to the methanolic extracts of the same plant, which presented activities comparable to that of BHT. The authors considered the absence of phenolic compounds in the oils responsible for such weak activity. α-Pinene, borneol, camphene and trans-caryophyllene were the main components of the oil [48]. For the *Psammogeton canescens* (DC.) vatke oil the authors [49] also attributed the weak activity of the oils to the presence of β-bisabolene, apiole, α-pinene and dill apiole, all non-phenolic compounds.

The antioxidant, antimicrobial and antispasmodic activities of *Origanum acutidens* (Hand.-Mazz) Ietswaart from the Turkish flora were evaluated [50]. Carvacrol was the major component of the essential oil. This oil presented antioxidant activity, but inferior to that of the reference substance used by the authors (BHT). In spite of the high percentage of carvacrol present in the oil, the inhibition percentage only reached 65%, in contrast to the 100% of BHT.

#### 3.1.4. Thiobarbituric acid reactive substances (TBARS)

This method measures the malondialdehyde (MDA) formed after lipid hydroperoxide decomposition (secondary products of oxidation), which forms a pink chromophore with thiobarbituric acid (TBA). This coloured complex, which absorbs at 532 nm, results in the condensation of TBA and malondialdehyde in an acidic environment. This method is not very specific, because 4-hydroxy-alkenals, 2,4-alkadienals and 2-alkenals, protein and sugar degradation products, amino acids, nucleic acids and anthocyanins are also able to react with TBA, forming a chromophore [5].

The essential oil of *Ageratum conyzoides* L., mainly constituted by precocene I and caryophyllene, possessed good capacity for preventing lipid peroxidation, using as lipid substrate liver homogenate [51]. The results were even better than those found for the reference BHA. The authors have also tested methanolic extracts of the same plants but the activities were about 100 times lower than those of the essential oils. The authors also hypothesise that the antiaflatoxigenic activity found for the essential oils may be partly due to their antioxidant activity [51].

Antioxidant activity of essential oils of five spice plants used in the Mediterranean diet was evaluated [52] through the thiobarbituric acid reactive species (TBARS) test using egg yolk as substrate. All essential oils tested (*Thymus vulgaris* L., [*Eugenia caryophyllus* (C. Spreng) Bull et Hare], *Origanum vulgare* L., *Salvia officinalis* L. and *Rosmarinus officinalis* L.) had antioxidant activity, but *Thymus vulgaris* oil presented the best activity, close to that verified for BHT. Terpinen-4-ol, γ-terpinene, *cis*-sabinene hydrate, linalool and *p*-cymene predominated.

The antioxidant activity of *Thymus* essential oils from Portugal were the target of study in [53]. The authors studied the capacity of preventing lipid peroxidation through the same method described by [52] and they found that *Thymus zygis* subsp. *zygis* and *Thymus zygis* subsp. *sylvestris* oils obtained from different regions of Portugal showed good antioxidant activities, even better than that of BHT. Carvacrol, thymol, *p*-cymene and γ-terpinene predominated in the oils, although in different percentages.

*Thymus marschallianus* Will. and *Thymus proximus* Serg. oils from China had lower capacity for preventing lecithin peroxidation than BHT, using TBARS method. Nevertheless, between the oils, that isolated from *Thymus proximus* had much higher activity. *p*-Cymene, γ-terpinene and thymol predominated in this ois, whereas in the other oil, only γ-terpinene and thymol dominated. According to these results, the authors [54] stressed the importance of *p*-cymene in the antioxidant activity of *Thymus proximus*, besides thymol and γ-terpinene.

Among seven Himalayan Lauraceae species, only the essential oils of *Dodecadenia grandiflora* and *Lindera pulcherrima* were able to inhibit lipid peroxidation, using liver homogenate as lipidic substrate. These oils, along with *Persea gamblei*, were already reported as being potent inhibitors of linoleic oxidation measured through the β-carotene bleaching test [44]. Such activities were independent of the presence of phenolic compounds in the essential oils.

The evaluation of the antioxidant activities of essential oils from *Capparis spinosa* L. and *Crithmum maritimum *L. from Dalmatia (Croatia) by different methods, showed that using TBARS method, the oils presented at 1 g/L (the highest concentration tested) lower ability to inhibit lipid peroxidation than BHA, but close to that of BHT. They also found that the activity was dose-dependent. The differences of activity of both samples was not significant, although the differences in the chemical composition of the oils were. *Capparis spinosa* oil was predominantly constituted by methyl isothiocyanate, whereas sabinene and limonene were the main components of *Crithmum maritimum* oil [55].

*Amomum tsao-ko* Crevost & Lemairé is a zingiberaceous plant called ‘Caoguo’ or ‘Tsao-ko’, which is widely distributed in the south-west of China. As some reports have related cytotoxicity with antioxidant activity and the essential oil studied by the authors had substantial anticancer activity, [56] assayed the antioxidant activity of their samples using diverse methods, being one of them the TBARS method. The lipidic substrate was constituted by liposomes containing lecithin, and the induction of peroxidation was performed with ferrous sulphate. They found weak antioxidant activity, as expected by the authors, due to the low phenolic content of the oils. The main component was 1,8-cineole. The absence of phenolic compounds related with weak antioxidant activity is not in accordance with those reported by [44,55], which found potent and moderate activity with oils presenting furanodiene, germacrene D, furanodienone, curzerenone, and sabinene and limonene, respectively. In spite of the different composition of the oils it is also important to stress that this difference of activities may also be attributed to the different lipidic substrate used in both works.

Aerial parts and seeds of *Foeniculum vulgare* Mill. oils possess different chemical compositions, nevertheless in what concerns the antioxidant activity, the authors in [57] found that for higher concentrations of essential oils (>750 mg/L for the aerial parts and > 1,000 mg/L for seeds) a decrease of the activity was observed, suggesting that high concentrations of essential oils possess a pro-oxidant activity, independent of their richness in *trans*-anethole (aerial parts) or methyl chavicol (seeds).

Suanarunsawat *et al.* (2010) [58] have investigated the anti-hyperlipidemic and antioxidant activities of essential oils extracted from *Ocimum sanctum* L. leaves in rats fed with high cholesterol diet. Eugenol and methyl eugenol were the most important components in the essential oils. The oils were able to decrease the high levels of TBARS either in cardiac or liver tissues, protecting them against stress induced oxidation.

Malonaldehyde can also be measured through gas chromatography with a nitrogen phosphorous detector, after derivatization of malonaldehyde to 1-methylpyrazole with *N*-methylhydrazine. This method was followed by [43] that demonstrated the oils of *Thymus vulgaris* and *Eugenia caryophyllus* possessed the strongest activity, even comparable to that of α-tocopherol. However, in the same work the authors also reported that at higher concentrations of thyme oil, such capacity decreased. Basil oil, that was considered a good antioxidant when the activity was measured through the conjugated diene assay, did not stand out in this test, in which was only considered as possessing moderate activity. Such results may reveal that basil is only able to prevent the primary oxidation of lipids.

#### 3.1.5. Aldehyde/carboxylic acid assay

This assay is convenient for evaluating the effects of antioxidants against slow oxidation phenomena occurring over prolonged periods of time, as shown in the shelf life of foods [59]. The authors [43] using this method showed that the oils of *Thymus vulgaris, Eugenia caryophyllus*, *Cinnamomum zeylanicum* Blume., *Ocimum basilicum* L. and *Illicium verum* Hook. f. were able to prevent the oxidation of hexanal to hexanoic acid promoted by heat and pure air introduced in a sealed system, after 40 days of storage. The decrease of hexanal was followed by gas chromatography coupled to a flame ionisation detector. Such results may demonstrate the low importance of phenolic compounds to prevent hexanal oxidation, since *Illicium verum* oil was mainly cosnstituted by anethole [43].

#### 3.1.6. Formic acid measurement

The Rancimat method is an automated test that measures the conductivity of low molecular weight fatty acids (formic acid) produced during the auto-oxidation of lipids at 100 ºC or above [5]. The antioxidant activity of essential oils of five spice plants used in the Mediterranean diet that was evaluated through the TBARS method [52] was also evaluated through the Rancimat method in which the lipid substrate was lard. The system was heated at 120 ºC and an air flow of 20 L/h was constantly blown into the mixture. The end of the induction period was characterized by the sudden increase of water conductivity, due to the dissociation of volatile carboxylic acids. Their results showed that the oils of *Thymus vulgaris* and *Origanum vulgare* L. showed the best antioxidant activity index, as determined by the Rancimat method, but less than the activity of BHT (synthetic standard). Carvacrol was the main component of the *Origanum vulgare* oil. In this case, an oil rich in phenolic components (oregano) and the other one (thyme) without great levels of these components presented similar activities, in contrast to that reported for the TBARS, in which thyme possessed the best activity [52]. This is a good example in which diverse methods of antioxidant evaluation may give rise different results, showing therefore the importance of using several methods to evaluate the enatioxidant activity of samples.

Supercritical fluid extraction of the volatile oil from *Thymus vulgaris* L. aerial parts and the correspondent composition was compared with those of the essential oil isolated by hydrodistillation. The main components in both cases were *p*-cymene, γ-terpinene, linalool, thymol and carvacrol. The percentages changed according to the method of extraction used, nevertheless *p*-cymene and thymol dominated in both cases. The great difference was found to be the presence of thymoquinone in the volatile oil obtained through the supercritical fluid extraction. The presence of this component could be the responsible for the highest antioxidant activity assessed by Rancimat test, in which the lipidic substrate was sunflower oil [60].

It is also noteworthy to stress the difference in the chemical composition of *Thymus vulgaris* oils reported by [52,60], showing therefore the importance of the evaluation of the chemical composition of samples.

### 3.2. Free radical scavenging ability

For measuring free radical scavenging ability, the methods are grouped in two groups, according to the chemical reactions involved: hydrogen atom transfer reaction-based methods and single electron transfer reaction-based methods [5].

#### 3.2.1. 2,2-Diphenyl-1-picrylhydrazyl (DPPH) assay

The most commonly used DPPH assay is simple and highly sensitive. DPPH is commercialised in the radical form due to its stability. This radical shows a strong absorption maximum at 517 nm (purple). In the presence of antioxidants, the colour turns from purple to yellow. Therefore the sole equipment needed for the assay is a UV-Vis spectrophotometer.

Initially, DPPH radical was thought to be reduced to the corresponding hydrazine when it reacted with the donating hydrogen substances. However, more recent studies have shown that what occurs is mainly a fast electron transfer from the sample to DPPH radical. The abstraction of hydrogen from the sample by DPPH radical is marginal, because it occurs very slowly and depends on the hydrogen-bond accepting solvent. Methanol and ethanol, solvents generally used for antioxidant ability assays, are strongly hydrogen bond-accepting, therefore the hydrogen-abstracting reaction occurs very slowly [5]. 

Due to its simplicity and sensitivity, some authors only use DPPH method for evaluating the antioxidant activities of essential oils. Recently, in our bibliographic research, some examples could be found [61,62,63,64,65,66,67,68,69,70,71,72,73,74,63,64]. Sometimes, the decision of only using this method can be attributed to the low antioxidant activity detected, therefore the authors consided it unnecessary to pursue other methods. One example is the case reported in [61], in which the authors found low antioxidant activities of *Commiphora ornifolia *(Balf. f.) Gillett and *Commiphora parvifolia *Engl. oils even at high concentrations. According to these results along with the chemical composition of the oils, in which phenolic compounds were absent, the authors probably considered it unnecessary to use other methods. Another example is the oil of leaves of *Olea europaea* L. cv. Chemlali from Tunisia that also possesses low activity when compared to BHT [70], because the main components were α-pinene and 2,6-dimethyloctane, that is, non-phenolic compounds.

Nevertheless, there are other authors that consider it enough to test using only one method (DPPH) when good antioxidant activity values are found [62,66,67,68,70]. For example, the authors in [62] found high antioxidant activities of the essential oils of *Eucalyptus camaldulensis* Dehnh. growing wild in different localities of Sardinia (Italia). The activities changed according to the chemical composition, neverthless generally constituted by *p*-cymene, 1,8-cineole, β-phellandrene, spathulenol and cryptone in different proportions according to the harvesting place and period. The essential oil of *Majorana hortensis* L. had appreciable antioxidant activity probably ascrible to carvacrol with positive synergism with other components [66]. *Citrus maxima* Burm. and *Citrus sinensis *(L.) Osbeck also had considerable antioxidant activities, despite the chemical differences of their essential oils. Whereas *Citrus sinensis* oils was predominantly constituted by limonene, in *Citrus maxima* oil, several components could be identified in significant amounts in the oil (limonene, *E*-citral, *Z*-citral and 3,3-dimethyl-1-hexene [67]. *Heracleum pastinacifolium* and *Heracleum persicum* were described as possessing moderate antioxidant activity, which main components were myristicin and *trans*-amethole, respectively [68]. Three Mexican *Lippia graveolens* Kunth. oils with different chemical compositions as well as their microcapsules were evaluated in terms of antiradical activities. The authors concluded that microencapsulation increased the anti-radical activity from fourfold to eightgold [69]. Diverse oils obtained from *Origanum vulgare* L. subsp. *glandulosum *(Desf.) collected at different locations of Tunisia showed different but always good abilities for scavenging DPPH radicals. Such results depended on the percentage of phenolic compounds (thymol) present in the oils [71]. In spite of the fact some authors consider that the flower oils of *Retama raetam* (Forssk.) Webb cultivated in Tunisia presented good antioxidant activity, as measured through the DPPH method, the sole method used by the authors, whereby the IC_50_ value was fortyfold superior to that of the reference BHT, nevertheless the apparent relative good activity reported by the authors could be attributed to the relative high percentage of monoterpenes present in the essential oils [72]. A good activity was found by Saei-Dehkordi *et al. *(2010) [74] for the root oil of *Ridolfia segetum* (L.) Moris from Tunisia, because the IC_50_ values were close to that of BHT. Such antiradical activity could be attributed to the high amounts of two phenyl-propanoids, dillapiole and myristicin [60].

The DPPH method was also the sole one used by [63], although the authors also performed the DPPH method but coupled to TLC (thin layer chromatography), that allows determination of which compounds are resposnsible for antioxidant activity. Using two tests based on the same principle but using diverse methodologies (spectrophotometer and TLC), the authors were able to find the compounds responsible for such activities after separation by TLC. The authors identified 1,8-cineole and methyl eugenol as the components responsible for the moderate antioxidant activity of the essential oils of *Myrtus communis* L.. The same procedure was followed by other authors [64] for the oils of *Aniba panurensis* (Meisn.) Mez, *Aniba rosaeodora* Ducke and *Licaria martiniana* (Mez) Kostern., from Brazil. The IC_50_ values were generally >1,000 μg/mL, substantially superior to that of quercetin, the reference used by the authors. Such low activity was attributed to the absence or to the very low concentrations of some compounds responsible for antioxidant activity – β-caryophyllene along with phenolic compounds. Once again, and as reported above, such results might be enough for the authors to decide not to do other assays.

Saleh *et al.* (2010) [65] have conducted a comparative evaluation of the antioxidant properties of 248 essential oils by the spectrophotometer method of DPPH along with DPPH/TLC. Only 17 species possessed effective antioxidant activity, mainly belonging to the Lamiaceae family. The active compounds detected by DPPH/TLC method included phenols, non-phenols, oxygenated or non-oxygenated compounds.

In spite of the fact that some authors choose only one method for evauating the antioxidant activity, the great majority prefers to check at least two assays. The combinations may include assays for evaluating the ability for inhibiting lipidic peroxidation through the methods reported above and other ones not included in this text and tests regarding the ability for scavenging free radicals.

Some of the authors that had evaluated the ability of essential oils to prevent lipid peroxidation by the TBARS method, also had evaluated the capacity for scavenging free radicals, using the DPPH assay [44,54,51,52,53,56,57].

Not always the best activity found in the TBARS assay also corresponds to the best ability to scavenge DPPH radicals. Such was found [44,51]. The essential oils of *Ageratum conyzoides* or *Amomum tsao ko* presented better ability to prevent lipid peroxidation than for scavenging free radicals. *Ageratum conyzoides* methanolic extracts that had low capacity for preventing lipid oxidation, in the DPPH assay were revealed to be more effective than the essential oil [51]. On the other hand *Lindera pulcherrima*, *Dodecadenia grandiflora* and *Dodecadenia gamblei *presented the best inhibition of lipid peroxidation determined through the TBARS method as well as in the β-carotene bleaching test, but in the DPPH method, only *Dodecadenia grandiflora *was a powerful DPPH radical scavenger [44]. Another example was that reported [52] for *Thymus vulgaris* and *Eugenia caryophyllus* oils. In this assay, the authors found that the *Thymus vulgaris* oil had the best activity against lipid oxidation, whereas in the DPPH method, the authors found that *Eugenia caryophyllus *oil was the most potent scavenger of DPPH free radicals, even better than the standards ascorbic acid and BHT. The same authors also used the Rancimat test, and in that ase, the best oil was that of *Origanum vulgare*, although worse than that of the reference BHT. The non-polar fraction and essential oil of *Hymenocrater longiflorus* possessed great ability to inhibit the bleaching of β-carotene, nevertheless the results differed when the DPPH method was used. In this case, the essential oil was the only one that had the worst capacity for scavenging these free radicals [47]. Independent of the plant part of *Myrtus communis* var. *italica* L. used and the antioxidant method used, the methanolic fractions were always better antioxidants than the essential oils [46]. This is one example in which in both methods the results maintained in contrast to those reported until now.

As already reported above, there are researchers that prefer to evaluate the antioxidant activity of the essential oils through their ability to scavenge free radicals. There are several free radicals that can be used; nevertheless the fundamental principles involved may be the same.

#### 3.2.2. Trolox® equivalent antioxidant capacity (TEAC) or ABTS [2,2-Azinobis(3-ethylbenzo-thiazoline-6 sulphonic acid)]

This method relies on the reduction of the blue-green cation radical of ABTS^+^^•^, by measuring the reduction of the radical cation as the percentage of inhibition of absorbance at 734 nm. The absorbance of the reaction mixture of ABTS and an antioxidant is compared to that of the Trolox^®^ standard, and the results are expressed in terms of Trolox^®^ equivalent antioxidant capacity (TEAC) [59].

As reported for the DPPH method, there are also some authors that only used the ABTS method for evaluating the antioxidant activity of essential oils [75,76]. Ennajar *et al.* (2010) [75] studied the influence of organ, season and drying method on chemical composition and the ABTS-scavenging ability of *Juniperus phoeniceae* L.. They found the highest and the lowest activities in the same part of the plant (berries). Considering the drying method used, oven-drying was the best method to obtain an important activity for leaf and berry oils, followed by sun-dried essential oils and shade-dried samples. Such variability seemed to be related to the chemical composition of the oils, wich having a similar profile, the main components were present in different proportions (α-pinene, δ-3-carene, α-terpineol, β-myrcene, among other components). *Rosa canina* L. also possessed capacity for scavenging ABTS, nevertheless the harvesting place was determinant in such activity, which seemed to be related to the presence of high percentages of vitispirane [76]. 

However and generally we find other tests being utilized along with ABTS for evaluating free radical-scavenging capacity. For example, *Satureja intricata* Lange had ability for scavenging both DPPH and ABTS, whereas *Satureja obovata* Lag. did not possess such ability [77]. This behaviour could be attributed to the presence of thymol and its precursors *p*-cymene and γ-terpinene, along with borneol in *Satureja intricata*. The essential oils of *Artemisia herba-alba* already reported above as possessing little ability for inhibiting lipid peroxidation [45], when evaluated in terms of free radical scavenging, the oils continued to have weak activity. In this case and in contrast to other works [44,51], the essential oil of *Artemisia herba-alba* was not effective either against lipid oxidation nor as a free radical scavenger.

Lavander (*Lavandula angustifolia*) oil, as reported above, was the most effective against lipid peroxidation [42]. Concerning free radical-scavenging activity, lavender essential oil also exhibited the highest DPPH-scavenging activity, neverthelesss in the ABTS-scavenging activity, the best oils were those of *Mentha x piperita* L and *Boswellia carteri* Birdw. In the DPPH method, limonene had similar activity of that of *Lavandula angustifolia*, although linalool and linalyl acetate constituted the major components of this essential oil. Limonene predominating in the *Citrus x limon* (L.) Burm. f. oil did not icorrespond to the best activity.

#### 3.2.3. Ferric reducing/antioxidant power (FRAP) assay

When a Fe^3+^-TPTZ (2,4,6-tripyridyl-*s*-triazine) complex is reduced to the Fe^2+^ form by an antioxidant under acidic conditions, an intense blue color develops with an absorption maximum at 593 nm. The antioxidant effect (reducing ability) can be evaluated by monitoring the formation of a Fe^2+^-TPTZ complex with a spectrophotometer [59].

The antioxidant activity of *Pistacia atlantica* Desf. leaf oils from diverse origins of Algeria showed weak ability for scavenging DPPH free radicals, at least when compared to those of references (BHT and BHA). In contrast, the same oils had a higher antioxidant capacity relative to the antioxidant of reference ascobic acid when measured through the FRAP assay [78]. In the same assay, the authors also reported diverse activities depending on the harvesting place. The best activity was found in the oil obtained from Laghouat, mainly constituted by α-pinene + α-thujene, camphene and spathulenol.

By comparing the antioxidant activity measured by DPPH radical scavenging and ferric reducing power (FRAP) of *Zingiber officinale* Roscoe and *Cuminum cyminum* L. oils, El-Ghorab *et al.* (2010) [79] reported almost similar trends in both methods. *Cuminum cyminum*, mainly constituted by cuminal, γ-terpinene, pinocarveol,, carotol, α-pinene, sabinene, β-terpineol and linalool, presented the best activity.

*Eugenia caryophyllus* (clove) showed the highest ferric reducing capacity in terms of Trolox^®^ concentrations, such as in the DPPH method, in contrast to the TBARS method, in which *Thymus vulgaris* showed to be more effective [52]. According to the same authors, a significant linear correlation between FRAP values and total phenolic contents of the essential oils analysed were observed, except for the *Thymus vulgaris* essential oil.

*Amomum tsao-ko* oil with low antioxidant activity measured through the TBARS method [70], as reported above, also had weak ability for scavenging DPPH free radicals and low ferric reducing power, mainly due to the low phenolic content of the oils [56].

The antioxidant measurement by DPPH and FRAP methods showed that the essential oil of *Eugenia caryophyllus* exhibited higher activity than the volatile aglycone fraction obtained after enzymatic hydrolysis with β-glucosidase from bitter almonds of the extract and further extraction from the aqueous layer with *n*-pentane [80]. In the DPPH method, the antioxidant activity of clove oil was higher than the volatile aglycone fraction, although both samples have high eugenol content. This lower activity could be attributed and according to the authors to the antagonism among other constituents present in small amounts in this fraction. The reducing capacity of the essential oil was also higher than that of the volatile aglycone fraction. Nevertheless in this assay the reference eugenol had weak ability, whereas in the DPPH method it possessed considerable activity. Such a difference could be explained by the different polarity for FRAP method compared with DPPH method. “Water-like” solvents induce a dramatic decrease in the hydrogen atom-donating capability in comparison with ethyl alcohol used in DPPH method [80].

Seasonal qualitative and quantitative differences in composition and antioxidant activities of male and female leaf essential oils of *Pistacia atlantica* were observed. The main components of male leaf essential oil were α-pinene/α-thujene, spathulenol and bicyclogermacrene. The major component of female leaf essential oil was δ-3-carene. The seasonal variation showed that most of the main components of the oils reached theirs highest values in September. The highest antioxidant capacity to scavenge free DPPH radicals was reached in the month of June for male oils and during the months of September - October for the female oils. The high reducing power for male oil was detected during the month of June and for the female oil was in August. The antioxidant activity of the female oil was almost ten times higher than ascorbic acid in the FRAP assay [81].

#### 3.2.4. Reducing power

Another method to evaluate antioxidant ability is based on the reduction of Fe^3+^ to Fe^2+^ in which the yellow colour of the test solution changes to various shades of green and blue, depending on the reducing power of each sample. The presence of reducing agents causes the conversion of Fe^3+^/ferricyanide complex to the ferrous form that may be followed at 700 nm due to the formation of Perl’s Prussian blue Fe_4_[Fe(CN)_6_]_3_. Increasing absorbance at 700 nm indicates an increase in reductive ability [44]. Several authors along with other antioxidant activity assays also tested the reducing power of the essential oils of Himalayan Lauraceae species [44], *Myrtus communis* var. *italica* [46], *Hymenocrater longiflorus* [47], *Origanum onites* L. [82] and *Psammogeton canescens* [49]. In this case, the antioxidant activity measured by the β-carotene bleaching and DPPH tests showed weak activity. The authors attributed such activity to the low concentration of phenolic compounds present in the essential oils, nevertheless the reducing power of the same samples is similar to that of the reference ascorbic acid, mainly at higher concentrations [49].

#### 3.2.5. Chelating activity

One of the possible mechanisms of the antioxidative action is the chelation of transition metals. Transition metal ions can stimulate lipid peroxidation by participating in the generation of initiating species and by accelerating peroxidation, decomposing lipid hydroperoxides into other components which are able to abstract hydrogen, perpetuating the chain of reaction of lipid peroxidation [52].

One method usually used for the determination of chelating activity uses ferrozine, which can quantitatively from complexes with Fe^2+^. In the presence of other chelating agents, the complex formation is disrupted, giving rise to a decrease of the red colour of the complex ferrozine-Fe^2+^. Measurement of the rate of colour reduction therefore allows estimation of the chelating activity of the coexisting chelator [46].

Essential oils of *Myrtus communis*, *Thymus marschallianus* and *Thymus proximus* generally did not present chelating activity [46,54]. The sole exception was the essential oils of flowers of myrtle. The authors [46] explained this activity to the presence of eugenol and methyl eugenol in its essential oil, whereas these two components only belong to a minor fraction of leaf and stem essential oils. These dihydroxylated compounds would be necessary to form chelated Fe^2+^. Leaf and stem oils had as main components 1,8-cineole and terpinen-4-ol, monohydroxylated compounds that would be unable to chelate ferrous ions [46]. Concerning the essential oils of spice plants used in a Mediterranean diet, all of them were able to chelate Fe^2+^ and did so in a concentration-dependent manner [52]. At all concentrations assayed *Rosmarinus officinalis* L. and *Salvia officinalis* essential oils showed the highest values for chelating Fe^2+^. All the oils studied were better chelators of Fe^2+^ than ascorbic acid and BHT, used as references [52].

#### 3.2.6. Hydroxyl radical scavenging

Among the oxygen radicals, hydroxyl is the most reactive one, inducing severe damage to the adjunct biomolecules. There are several ways to ascertain the ability to form hydroxyl radicals. One of them is the deoxyribose test. This method includes a mixture of ferric chloride (FeCl_3_) and ethylenediamine tetraacetic acid (EDTA), which in the presence of ascorbic acid forms Fe^2+^-EDTA, and oxidized form of ascorbic acid. After addition of hydrogen peroxide (H_2_O_2_), Fe^3+^-EDTA and HO^•^ are formed. This is the so-called Fenton reaction, which generates the highly reactive hydroxyl radical (Fe^2+^ + H_2_O_2_ → Fe^3+^ + HO− + HO^•^). Hydroxyl radicals that are not scavenged by any component of the mixture attack the deoxyribose and degrade it into several fragments. Some of these fragments are capable of reacting with thiobarbituric acid after heating and in an acidic pH, originating a pink pigment that can be quantified by spectrophotometry [5]. There are other methods that did not use deoxyribose but other components such as benzoic acid or safranine. Using these methods several authors have evaluated the hydroxyl radical scavenging ability of essential oils [51,53,54,57,83,84].

The ability to scavenge hydroxyl radicals of the essential oils of *Thymus marschallianus* and *Thymus proximus* were evaluated using the safranine method and the authors reported that the latter oil was more effective than the former. The activity of both oils were dose-dependent and they were mainly constituted by thymol, *p*-cymene and γ-terpinene [54].

Patil *et al.* (2010) [51] have compared two methods (deoxyribose and benzoic acid) on the hydroxyl radical scavenging ability of *Ageratum conyzoides*, mainly constituted by the phenolic chromenes precocene I and precocene II, and they obtained different responses according to the method used. In the benzoic acid hydroxylation method, the essential oil presented better activity than in the deoxyribose method. The authors explained such results to the relative reactivity of hydroxyl radical towards the substrates deoxyribose and benzoic acid.

Among Portuguese *Thymus* species collected in different places of this country, *Thymus camphoratus* Hoffmanns. & Link, *Thymus caespititius* Brot., and *Thymus capitellatus* Hoffmanns. & Link oils from different origins were significantly more effective in scavenging hydroxyl radicals than the remaining samples. Borneol, camphor, α-terpineol, 1,8-cineole, camphene, α-pinene and *p*-cymene constituted the major compounds of these essential oils in different relative amounts. The oils in which thymol or carvacrol predominated did not present significant activities, which according to the authors [53] indicated that these phenolic compounds are not determinant in the ability for scavenging hydroxyl radicals.

The oils from young and mature leaves of *Ageratum scoparia* Waldst. & Kit. displayed a high degree of hydroxyl radical scavenging activity. However, the scavenging activity of principle constituents, β-myrcene and *p*-cymene, was very less compared to that of oils. The same authors [83] also evaluated the antioxidant activity of the samples by the DPPH method and also reported less activity of the main constituents of the oils than the essential oils, nevertheless the mature leaf oils were more effective as antioxidants than young leaf oils.

The mature fruits of *Athamanta turbith* ssp. *hungarica* and *Athamanta turbith* ssp. *haynaldii* are rich in essential oils, myristicin being the main component of both oils [84]. The capacity for scavenging hydroxyl radicals was evaluated and the authors found that both oils had similar ability. When the activity was evaluated by the DPPH method, the authors reported that *Athamanta turbith* ssp. *hungarica* was the most effective, albeit with less ability than the references used (quercetin and ascorbic acid) [84].

The capacity of the essential oils obtained from the aerial parts or from the fruits of *Foeniculum vulgare* for scavenging hydroxyl radicals was always less than 50%, at the concentrations assayed (100-2,000 mg/mL) [57]. Mannitol, the reference used in this assay, showed a similar scavenging percentage at ≈2,000 mg/mL. Only higher concentrations presented percentages >50%.

#### 3.2.7. Superoxide anion scavenging activity

Xanthine oxidase is a dehydrogenase enzyme that transfers electrons to nicotinamide adenine dinucleotide (NAD^+^), reducing it to NADH, and oxidizes xanthine or hypoxanthine to uric acid. Nevertheless, under stress conditions, the dehydrogenase is converted to an oxidase enzyme and, under these conditions, the enzyme reduces oxygen instead of NAD^+^. On this way, there is a reduction of dioxygen to superoxide anion and hydrogen peroxide. Superoxide anion can be generated by this system (the hypoxanthine - xanthine oxidase) or using a non-enzymatic reaction. In this case, superoxide anion is generated through the reaction of phenazine methosulphate in the presence of NADH and dioxygen. In both cases, superoxide anion reduces nitro-blue tetrazolium (NBT) into formazan which is followed spectrophotometrically [5].

The superoxide anion scavenging activity of Portuguese *Thymus* oils were evaluated by the enzymatic hypoxanthine/xanthine oxidase system [53]. From the 28 essential oils evaluated, only *Thymus zygis* ssp. *sylvestris* and *Thymus capitellatus* collected in diferent places of Portugal had superoxide anion scavenging activity >50%. In *Thymus zygis* ssp. *sylvestris* prevailed thymol or carvacrol, whereas in *Thymus capitellatus* oils, borneol and 1,8-cineole dominated. Such results seem to indicate that the phenolic compounds are not the only ones able to scavenge superoxide anion, as already reported for hydroxyl radical scavenging [53].

## 4. Essential Oils as Anti-Inflammatory Agents

Inflammation is a normal protective response induced by tissue injury or infection and functions to combat invaders in the body (microorganisms and non-self cells) and to remove dead or damaged host cells [85].

In the inflammatory response there is an increase of permeability of endothelial lining cells and influxes of blood leukocytes into the interstitium, oxidative burst, and release of cytokines [interleukins and tumor necrosis factor-α (TNF-α)]. At the same time, there is also an induction of the activity of several enzymes (oxygenases, nitric oxide synthases, peroxidases) as well as the arachidonic acid metabolism. In the inflammatory process there is also the expression of cellular adhesion molecules, such as intercellular adhesion molecule (ICAM) and vascular cell adhesion molecule (VCAM) [22].

In addition to the ability of some essential oils to scavenge free radicals, there is also evidence that some essential oils possess anti-inflammatory activity. For example, chamomile essential oil has been used for centuries as an anti-inflammatory and also for alleviating the symptoms associated with eczema, dermatitis and other pronounced irritation [17]. However, there are other examples of essential oils (eucalyptus, rosemary, lavender, millefolia) along with other plants (pine, clove and myrrh) that have been used as mixed formulations as anti-inflammatory agents [86].

Carrageenan-induced mouse paw oedema is frequently used to determine the anti-inflammatory activity of diverse bioactive compounds such as plant extracts and essential oils [87,88,89,90,91,92,93,94,95]. If this method allows screening the anti-inflammatory of samples, very little information is given about its mechanism.

The anti-inflammatory activity of essential oils may be attributed not only to their antioxidant activities but also to their interactions with signalling cascades involving cytokines and regulatory transcription factors, and on the expression of pro-inflammatory genes.

### 4.1. Effects on the arachidonic metabolism

Arachidonic acid is a polyunsaturated fatty acid that is released from cell membranes by phospholipase A_2_ under the stimulus of several factors associated with inflammation. The same fatty acid is metabolised by cyclooxygenase (COX) and lipoxygenase (LOX) pathways in different eicosanoids such as prostaglandins (PGs) and leukotrienes (LTs) in diverse cells and thromboxane A_2_ in platlets [22]. Two isoforms of COX, COX-1 and COX-2, have been reported. COX-1 is a constitutive enzyme whilst COX-2 protein is only slightly expressed in most normal mammalian tissues in response to physical, chemical and biological stimuli, including UV light exposure, dioxin and LPS (lipopolyssacharide) insult [96].

The prostaglandins, especially prostaglandin E_2_ (PGE_2_) amplify the pain mechanism and enhance vascular permeability whereas the leukotrienes contract the smooth muscles of blood vessels, enhance vascular permeability and mediate proinflammatory and allergic responses [97].

**Figure 1:**
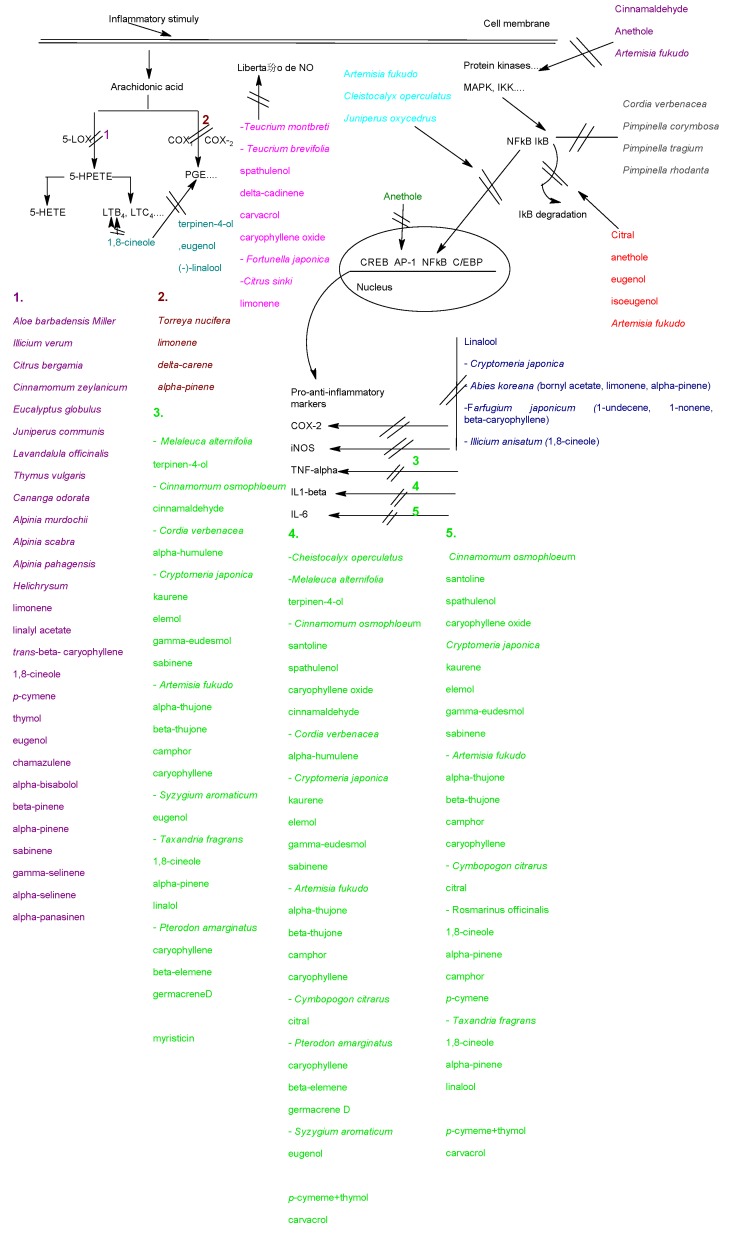
Essential oils and their main components in the modulation of the anti-inflammatory response. The pathways are described in the text together with the examples of essential oils and components.

Figure 1 represents the sites where essential oils or their main components have been detected as acting anti-inflammatory agents.

Aloe vera (*Aloe barbadensis *Miller), anise star (*Illicium verum *Hook f*.*), bergamot (*Citrus aurantium *subsp.* bergamia *(Risso) Wight & Arn*.)*, cinnamon leaf (*Cinnamomum zeylanicum *Blume.), eucalyptus (*Eucalyptus globulus* Labill.), juniperus berry (*Juniperus communis* L.), lavender (*Lavandula officinalis* Chaix & Kitt), thyme (*Thymus vulgaris* L.) and ylang-ylang [*Cananga odorata (Lam.) Hook. F et Thomson*] essential oils, in which limonene, linalyl acetate, β-*trans*-caryophyllene, 1,8-cineole, *p*-cymene, thymol and eugenol predominated, showed strong lipoxygenase inhibitory effects [43]. More examples of 5-lipoxygenase inhibitors included some *Salvia* essential oils from South Africa. The authors attributed such activities to the presence of 1,8-cineole, α-pinene and β-caryophyllene in the essential oils [98]. The same major components present in the essential oils of four indigenous South African *Helichrysum* species were also reported to inhibit 5-lipoxygenase [99]. Chamazulene and α-bisabolol are examples of other components, present in the essential oil of chamomile, with anti-inflammatory activities partly due to the inhibition of leukotriene synthesis since they appear to be good 5-lipoxygenase inhibitors [17]. The essential oils of leaves and rhizomes of *Alpinia murdochii* Ridl., *Alpinia scabra* (Blume) Náves and leaves of *Alpinia pahangensis* Ridl. also showed to be good 5-lipoxynease inhibitors. β-Pinene, α-pinene and sabinene were the main components of the leaf oils, whilst the sesquiterpenes γ-selinene, α-selinene and α-panasinsen predominated in the rhizome oils [100]. 

The oils *Torreya nucifera* Siebold et Zucc. oil, mainly constituted by limonene, δ-3-carene and α-pinene, was a COX-2 selective inhibitor having significant inhibitory effects on PGE_2_ production [101]. 1,8-Cineole, a terpene oxide, present in many essential oils was shown to inhibit leukotrienes (LTB_4_) and prostaglandins (PGE_2_), both pathways of arachidonic acid metabolism [102].

### 4.2. Effects on cytokines production

Interleukin-1β (IL-1β) and tumor necrosis factor-α (TNF-α) are two important pro-inflammatory cytokines with a relevant role in the inflammatory disorders. The major source of TNF-α is the cells of monocyte/macrophage lineage although T lymphocytes, neutrophils and mast cells also produce this pro-inflammatory cytokine. IL-1β is produced by a variety of cells types, including monocytes, macrophages, fibroblasts and endothelial cells [103]. The production of the pro-inflammatory cytokines may be induced by lipopolysaccharide (LPS) that is an endotoxin localised on Gram negative bacteria cell walls capable to activate macrophages, stimulating the production of inflammatory cytokines [104]. However, Gram positive bacteria may also contribute to the stimulation in the production of cytokines. Lipoteichoic acid (LTA) represents a class of amphiphilic molecules anchored to the outer face of the cytoplasmic membrane in Gram positive bacteria that causes cytokine induction in mononuclear phagocytes and a synergism with peptidoglycan (PG) has been described resulting in higher cytokine levels [105].

The essential oil of *Cheistocalyx operculatus*, which chemical composition was not determined, significantly inhibited lipopolysaccharide (LPS)-induced secretion of IL-1β and TNF-α cytokines in RAW 264.7 cells, a mouse macrophage-like cell line [103]. However, and some years earlier, some authors had already reported that tea tree oil (*Melaleuca alternifolia *Cheel.), mainly its major component (terpinen-4-ol), was able to suppress the production *in vitro* of TNF-α and IL-1β, as well as IL-8, IL-10 and PGE_2_ by LPS-activated human blood monocytes [106]. Six years later, Caldefie *et al. *(2006) [107] also reported the capacity of the essential oil of that species for suppressing IL-2 pro-inflammatory cytokine and stimulating the secretion of the anti-inflammatory IL-4 and IL-10 cytokines in human peripheral blood mononuclear cell (monocytes and lymphocytes) stimulated by the lectin phytohemagglutinin (PHA) from *Phaseolus vulgaris* L.. Also using PHA from *Phaseolus vulgaris* for stimulating mononuclear cells, Hammer *et al. *(2000) [108] reported that the essential oils of *Taxandria fragrans* (J. R. Wheller & N. G. Marchant) J. R. Wheeler & N. G. Marchant, comb. nov., mainly constituted by 1,8-cineole, α-pinene and linalool, decreased the production of the cytokines TNF-α and IL-6.

The ability to inhibit IL-1β and IL-6 production but not TNF-α was attributed to 1,8-cineole, santoline, spathulenol and caryophyllene oxide, major constituents of the essential oil of *Cinnamomum osmophloeum* Kaneh. leaves [109]. *Rosmarinus officinalis* L. oil, mainly constituted by 1,8-cineole along with α-pinene, camphor and *p*-cymene was only able to reduce the pro-inflammatory IL-6 production in the mouse colon in which colitis was induced by 2,4,6-trinitrobenzene sulphonic acid (TNBS), not suppressing IL-1β [110].

Cinnamaldehyde, isolated from an essential oil produced from the leaves of *Cinnamomum osmophloeum*, was reported to inhibit the secretion of IL-1β and TNF-α within LPS or lipoteichoic acid (LTA) stimulated murine J774A.1 macrophages. Cinnamaldehyde also suppressed the production of these cytokines from LPS stimulated human blood monocytes derived primary macrophages and human THP-1 monocytes [111].

*Cordia verbenacea* D. C. essential oil significantly reduced TNF-α levels but not IL-1β production in the subcutaneous tissue of the rat paw injected with carrageenan [112]. Some authors showed that from the essential oil of *Cordia verbenacea*, only α-humelene was able to reduce significantly the increase of TNF-α and IL-1β levels in the subcutaneous tissue of the rat paw after LPS treatment [113] or in carrageenan-injected rats, whereas (-)-*trans*-caryophyllene diminished only TNF-α release [114].

Investigating the effect of *Cryptomeria japonica* D. Don essential oil on the LPS-treated RAW 264.7 cells, through enzyme immunoassay, some researchers reported the remarkable inhibition of IL-1β, IL-6 and TNF-α by that oil [115]. In this essential oil, kaurene, elemol, γ-eudesmol and sabinene prevailed.

α-Thujone, β-thujone, camphor, and caryophyllene were the main components of *Artemisia fukudo *oil that were able to inhibit the release of TNF-α, IL-1β, and IL-6 in LPS-treated RAW 264.7 cells [116].

The mixture of stereoisomers geranial (*E*-isomer) and neral (*Z*-isomer), known as citral, constitutes the main components of *Cymbopogon citratus* (DC) Stapf. (lemongrass) essential oil. This essential oil revealed to be capable to suppress IL-1β and IL-6 in LPS-stimulated peritoneal macrophages of normal mice [117]. The same authors attributed similar properties to the eugenol, the main component present in the essential oil of *Syzygium aromaticum* (L.) Merr. et Perry (clove) [118]. Other authors also found that eugenol on human macrophages (U937) under the stimulation of LPS blocked the release of IL-1β, TNF-α and PGE_2_ [119]. Whether some essential oils are able to inhibit the production of pro-inflammatory cytokines such as TNF-α, some of them, particularly lemongrass, geranium and spearmint and their main components (citral, geraniol, citronellol, and carvone), can also suppress TNF-α-induced neutrophil adherence responses [120]. According to these authors, those essential oils or their major components do not affect TNF-α but do affect the neutrophil function to suppress their adhesion. Therefore and concerning lemongrass and citral and as reported above, they can also suppress IL-1β and IL-6 pro-inflammatory cytokines [117]. In other work, Lin *et al.* (2008) [121] revealed that citral, the main component of the fruit essential oil of *Cinnamomum insularimontanum* Hayata, inhibits TNF-α in RAW 264.7 cells stimulated by LPS.

Among the 21 spices [allspice (*Pimenta officinalis* Lindl.), cardamom (*Elettaria cardamomum* Maton.), caraway (*Carum car*v*i* L.), gardenia (*Gardenia jasminoides* Ellis), cumin (*Cuminum cyninum* L.), clove [*Syzygium aromaticum* (L.) Merr. & L. M. Perry], laurel (*Laurus nobilis* L.), coriander (*Coriandrum sati*v*um* L.), fenugreek (*Trigonella foenum-graecum* L.), cinnamon (*Cinnamomum zeylonicum* Blume), sage (*Sal*v*ia officinalis* L.), celery seed (*Apium gra*v*eolens* L.), thyme (*Thymus *v*ulgaris *L.), chenpi (*Citrus unshiu* Markov.), nutmeg (*Myristica fragrans* Houtt), star anise (*Illicium *v*erum* Hook f*.*), paprika (*Capsicum frutescens* L.), fennel (*Foeniculum *v*ulgare* Mill.), black pepper (*Piper nigrum *L.), Japanese mint (*Mentha ar*v*ensis* Linn. var. piperascens Holmes), and rosemary (*Rosmarinus officinalis* L.)] studied by some authors to evaluate their hepatoprotective activity by feeding rats with liver damage caused by LPS plus D-galactosamine (D-GalN), only myristicin, the major component of nutmeg oil, presented a potent hepatoprotective activity by suppressing LPS/D-GalN induced enhancement of serum TNF-α concentrations and hepatic DNA fragmentation in mice. Therefore, the hepatoprotective activity of myristicin might be, at least in part, due to the inhibition of TNF-α release from macrophages, according to the same authors [122].

Animals (mice) pretreated with *Pterodon emarginatus* Vogel. oil presented a marked reduction of IL-1 and TNF-α levels after submitted to a single intrapleural injection of carrageenan [123]. *trans*-Caryophyllene, β-elemene and germacrene D were the major components present in the present oil.

The anti-inflammatory effects of diverse combination of thyme (*p*-cymene and thymol as main components) and oregano (carvacrol as major component) oils on mice with TNBS-induced colitis showed that some combinations lowered the amounts of IL-1β and IL-6 cytokines [124].

The inhibitory effect of some of the essential oils on the production of pro-inflammatory cytokines reported in this chapter seems to be mediated by suppressing gene expression of these cytokines. Such was described by some authors because they found that the essential oils studied significantly suppressed the protein and mRNA expression of the cytokines in stimulated cells, assuming therefore, that the inhibitory effect of these essential oils on the expression of the pro-inflammatory cytokines occurs mainly at transcriptional level [103,116,123,124]. Thyme essential oil, mainly constituted by *p*-cymene and thymol, only at high concentrations (5,000 mg/L) significantly inhibited total mRNA IL-1β expression in the mouse colon in which colitis was induced by TNBS, not inhibiting significantly IL-6 expression [125].

### 4.3. Modulation of pro-inflammatory gene expression

Nitric oxide (NO), prostaglandins and cytokines participate in inflammatory events. NO synthase (NOS) and cyclooxygenase (COX) catalise the formation of NO and prostaglandins, respectively. COX-2 and inducible NOS are considered as being inducible forms of these enzymes.

There are three isoforms of NOS: constitutive (cNOS), endothelial (eNOS) and the other is cytokine-inducible NOS (iNOS). Inflammatory mediators such as IL-1, TNF-α or LPS stimulate iNOS expression in rodent macrophages *in vitro. *NO production and/or iNOS expression are also induced by inflammatory mediators in a wide variety of mammalian cells, producing large amounts of NO for prolonged time periods [25]. iNOS catalises the production of large amount of NO from L-arginine and molecular oxygen, is mainly triggered and regulated by a series of signalling pathways including nuclear factor-κB (NF-κB) transcription factor and mitogen-activated protein kinases (MAPKs) [116].

The inhibition of activity or expression of COX-2 by some anti-inflammatory is due to the interference with the signalling mechanisms that regulate the COX-2 gene. In this gene, four transcription factors including NF-κB, CCAAT/enhancer-binding protein (C/EBP), activator protein 1 (AP-1) and CRE-binding protein (CREB) have been identified as regulators of COX-2 transcription [22,126]. NF-κB and MAPKs are of current interest as potential targets in therapies form diverse inflammatory symptoms.

NF-κB is a transcriptional regulator that consists of homo- and heterodimers of proteins (p65 or RelA, p50/p105, c-Rel, p52/p100 and RelB). NF-κB is maintained as a latent form in the cytoplasm of cells where it is complexed to IκB inhibitor protein. Seven members of the IκB family of proteins have been identified and includes IκB-α. Upon activation of NF-κB, IκB-α is phosphorylated by IκB kinases (IKK) leading to proteasome-dependent degradation of IκB, which allows a rapid translocation of NF-κB into the nucleus where it binds to DNA. The most predominant NF-κB dimmer activated is p65:p50. Translocation of p65:p50 to the nucleus results in the transcription of several proinflammatory genes, such as cytokines (TNF-α, IL-1β, IL-6) and inducible enzymes (iNOS and COX-2) [127,128].

In macrophages and other types of cells, LPS activates three subclasses of MAPKs: extracellular signal-regulated kinase (ERK), c-Jun-terminal kinase (JNK) and p38. Studies have revealed that some compounds are able to suppress proinflammatory cytokines and NF-κB, to inhibit the COX-2 and iNOS expression inhibiting some of these MAPKS [129,130,131].

Peroxisome Proliferator-Activated Receptor (PPARs) are ligand-activated transcription factors that belong to the superfamily of nuclear hormone receptors, which also includes receptors for vitamin D, vitamin A, thyroid hormone, bile acids, and steroid hormones. The PPAR subfamily comprises three isotypes, PPARα, PPARβ/δ, and PPARγ, which play various roles in lipid and carbohydrate metabolism, cell proliferation and differentiation, and inflammation [132,133].

The anti-inflammatory activities of PPARs are partially effectuated by inhibition of NF-κB. PPARs are able to attenuate NF-κB function either by interfering with the transcription activating capacity of the NF-κB complex or by regulating genes that suppress the activation of NF-κB. By physical interaction with components of the NF-κB complex, PPARα impairs binding of NF-κB to the DNA and subsequent activation of inflammatory genes. Secondly, PPARα activation results in increased expression of IκB, the inhibitory protein that prevents the transfer of NF-κB to the nucleus [134,135]. On the other hand, Inoue *et al.* (2000) [136] consider that COX-2 expression is regulated by a negative feedback loop, mediated through PPARγ, especially in macrophages, indicating that PPARγ participates in cell type-specific control of COX-2 expression.

Only the essential oils of *Teucrium brevifolia* Schreber and *Teucrium montbretii* Benth. ssp. *heliotropiifolium* (Barbey) Davis from a group of four studied effectively inhibited LPS-induced NO production in macrophage cell line RAW 264.7. Spathulenol and δ-cadinene predominated in *Teucrium brevifolia* whereas carvacrol and caryophyllene oxide dominated in *Teucrium montbretii*. Such components revealed to be of great importance on the inhibition of NO production and, therefore, on the inflammation inhibition, since the main components of the remaining *Teucrium* species were caryophyllene and 4-vinyl guaiacol in *Teucrium flavum* L., and carvacrol and caryophyllene in *Teucrium polium *ssp. *capitatum* (L.) Arcangeli [137].

The essential oils of *Fortunella japonica* var. *margarita* (GumGyul) and *Citrus sunki* (JinGyul), which limonene predominared in both types of oils, also markedly inhibited LPS-induced NO production in RAW 264.7 cells in a dose-dependent manner, indicating that they had anti-inflammatiry effects, according to the authors [138].

Incubation of RAW 264.7 cells with *Origanum ehrenbergii* Boiss. oil, in which thymol and *p*-cymene predominated, induced a significant inhibitory effect on the LPS-induced NO production. *Origanum syriacum* L. predominatly composed by carvacrol and thymol did not show such ability [139]. However, more examples of NO inhibition can be reported, for example the production of NO was also inhibited by the citrus peel waste essential oil in LPS-activated RAW 264.7 macrophages [140], or the oil of *Distichoselinum tenuifolium* (Lag.) Garcia Martin & Silvestre, mainly composed by myrcene, that significantly inhibited NO production stimulated by LPS in macrophages, demonstrating, therefore, *in vitro* anti-inflammatory properties [141].

PGE_2_ was only inhibited by LPS-stimulated macrophages J774.A1 cells when treated with high concentrations of (-)-linalool. The same was observed for the COX-2 expression. The same monoterpene inhibited NO release but did not inhibit the increase of iNOS expression indicating that the inhibitory activity of linalool was mainly attributed to the iNOS enzyme activity [142].

In addition to the effect of *Cryptomeria japonica* essential oil on the LPS-treated RAW 264.7 cells, which presented a remarkable inhibition of IL-1β, IL-6 and TNF-α, as reported above, the authors also detected an inhibition of NO production by reducing iNOS protein and mRNA iNOS expression as well as a diminution of PGE_2_ due to the reduction of COX-2 protein and COX-2 mRNA expression [115].

The inhibitory effects of *Abies koreana* Horstmann’s Silberlocke essential oil, mainly constituted by bornyl acetate, limonene and α-pinene, on pro-inflammatory mediators (NO and PGE_2_) were related to the modulation of iNOS and COX-2 expression, since the expression of iNOS and COX-2 mRNA and iNOS and COX-2 protein was reduced [143].

An inhibitory effect of *Farfugium japonicum* (L.) Kitamura essential oil, namely constituted by 1-undecene, 1-nonene and β-caryophyllene, on NO and PGE_2_ production in LPS-stimulated RAW 264.7 cells were found by Kim *et al.* (2008) [144], with a concomitant inhibition of iNOS and COX-2 mRNA expression, might explain the anti-inflammatory activity of this oil. The same authors in another work, and using the essential oil of *Illicium anisatum* L., mainly constituted by 1,8-cineole, also demonstrated its capacity for inhibiting NO and PGE_2_ production in LPS-stimulated RAW 264.7 cells, along with the decrease of iNOS and COX-2 proteins and iNOS and COX-2 mRNA expression [145].

Following carrageenan injection into the rat paw, the systemic treatment with α-humulene and (-)-*trans*-caryophyllene markedly inhibited the expression of both COX-2 and iNOS. The possible mechanisms involved were not performed by the authors [114].

α-Humulene and *trans*-caryophyllene, the main constituents of *Cordia verbenacea* inhibited the LPS-induced NF-kB activation and neutrophil migration in the rat paw, however unable to inhibit LPS-induced MAP kinase activation [113].

The essential oils of diverse *Pimpinella* species and their isolates were able to inhibit NF-kB transcription induced by phorbol myristate acetate (PMA) in SW1353 cells. However, they did not present similar activities. The most remarkable activity was reported for three *Pimpinella* species and five pure compounds: *Pimpinella corymbosa* Boiss., *Pimpinella tragium* Vill. ssp. polyclada (Boiss. et Heldr. Tutin), *Pimpinella rhodanta* Boiss. oils and 4-(2-propenyl)phenylangelate, 4-(3-methyl-oxiranyl)phenyltiglate, 4-methoxy-2-(3-methyl­oxiranyl)phenyl isobutyrate, 4-methoxy-2-(3-methyl-oxiranyl)phenylangelate and epoxypseudoisoeugenol-2-methylbutyrate [146]. These results also indicated the importance of a common structure (epoxyphenylpropanoid moiety) present in some of these pure compounds for the best anti-inflammatory activity.

Cinnamaldehyde-mediated inhibition of cytokines production found by Chao *et al. *(2008) [111], and reported above, may be, at least on part, due to the reduction of ROS release as well as of those of JNK and ERK within LPS stimulated J774A.1 macrophages.

The fruit essential oils of *Cinnamomum insularimontanum* Hayata and citral were revealed to possess significant inhibitory effects on NO production in LPS-stimulated RAW 264.7 cells. Citral exhibited an inhibitory activity in LPS-induced iNOS protein expression, not found for COX-2. The authors studied the effect of citral on NF-κB pathway for enlightening the mechanism of the citral-mediated inhibition of iNOS transcription. The authors concluded that citral could prevent the degradation of IκBα in LPS-induced inflammation and reduce the p50 NF-κB levels in nuclear fractions [121]. In another study, it was reported that in human macrophage-like U937 cells, citral suppressed both LPS-induced COX-2 mRNA and protein expression, dose-dependently. Moreover, citral induced the mRNA expression of the PPARα and the PPARγ, suggesting that citral activates PPARα andγ, and regulates COX-2 expression [147]. A same behaviour was observed for carvacrol. In human macrophage-like U937 cells, carvacrol suppressed LPS-induced COX-2 mRNA and protein expression, suggesting that carvacrol regulates COX-2 expression through its agonistic effect on PPARγ [132]. However, this property of carvacrol was not as remarkable as was that of citral [147].

*Artemisia fukudo* Makino essential oil, mainly constituted by α-thujone, β-thujone, camphor, and caryophyllene attenuates LPS-induced inflammation by suppressing NF-κB and MAPK activation in RAW 264.7 macrophages. The essential oil in LPS-induced inflammation act through the reduction of IκBα phosphorilation and blockage of NF-κB and translocation of p50 and p65 units to the nucleus. The same oil also suppressed the LPS-induced activation of phosphorilation of ERK, JNK and p38 MAPKs [116].

*Cleistocalyx operculatus* (Roxb.) Merr and Perry oil not only significantly inhibited LPS-induced secretion of pro-inflammatory cytokines, TNF-α and IL-1b, in RAW 264.7 cells, as reported above, but also suppressed the mRNA expression of TNF-a and IL-1b as well as blocked LPS-induced transcriptional activation of NF-κB and nuclear translocation of p65 subunit [103].

In a short report, Chainy *et al.* (2000) [148] described that anethole, eugenol and isoeugenol inhibited TNF-α-induced NF-κB activation in ML1-a cells. The same authors also reported that anethole inhibited the degradation of IκBα, blocking its phosphorylation in TNF-stimulated ML1-a cells. In the same cells and with TNF stimulation, anethole did not induce the phosphorylation of MAP kinase. Besides NF-κB and MAP kinase, anethole also blocked TNF-induced JNK and AP-1.

Early growth response factor-1 (Erg-1) is a transcription factor that plays a regulatory role in the expression of many important genes of inflammation (cytokine, cell adhesion molecules, and immunoreceptors). The human monocyte cell line THP-1 is often used as a model for tissue macrophages. Zhou *et al.* (2007) [149] showed that 1,8-cineole inhibited the Egr-1 synthesis and nuclear localization induced by LPS in THP-1 cells but did not affect the LPS-induced NF-κB expression in nuclei. Therefore, the authors concluded that 1,8-cineole may preferentially block the effect of Egr-1 by inhibiting the synthesis of Egr-1 and preventing Egr-1 nuclear internalization instead of NF-κB inhibition [149]. Some authors reported that α-pinene clearly inhibited the translocation of NF-κB/p65 protein into nuclei in LPS-stimulated THP-1 cells, through the increase of expression of IκBα protein [150,151].

The essential oil from the leaves of *Juniperus oxycedrus* L. of the Iberian flora, mainly constituted by α-pinene, showed a great inhibition of IL-1-induced NO production in the human chondrocytic cell line C-28/12. The same oil and a α-pinene-rich fraction reduced markedly IL-1-induced IκBα degradation and phosphorylation, NF-κB-DNA binding activity and NO production [152].

## 5. Conclusions

The antioxidant activity of essential oils has been target of investigation due to their potential as preservatives, cosmeceuticals or nutraceuticals in the food and cosmetic industries. The search for anti-inflammatory activities in essential oils has also increased during these last years. This work reports the antioxidant activities of essential oils which may act by preventing lipid peroxidation, scavenging free radicals or, and in very few cases, chelating metal ions. Some works also showed that the constituents of essential oil act synergistically because their main components when used as references have less activity than the essential oil. Therefore, synergism and antagonism studies must be developed.

Recently, Cabrera and Prieto (2010) [153] were able, for the first time, to show the application of artificial neural networks (ANN) on the prediction of the antioxidant activity of essential oils in two experimental in vitro models: in the DPPH and linoleic acid models. They used about 30 components from 80 that are described as possessing antioxidant activity and the results confirmed that this computational technique was reliable for predicting the antioxidant activity of essential oils from some of its components. Some of them are also reported in the present work (thymol, carvacrol, eugenol, *p*-cymene) among others. Nevertheless, the authors only tested two models. There are much more, those that allow evaluating the ability for scavenging ROS or RNS or chelating metals. In this way, the work is not finished and much more needs to be done.

According to the literature, it was possible to conclude that depending on the chemical compositon of the oils, they can act as anti-inflammatory affecting the arachidonic metabolism or the cytokines production, or on the modulation of pro-inflammatory gene expression.

The antioxidant and anti-inflammatory activities of the essential oils are well documented; nevertheless their uses can be hampered due to the chemical variability of the oils. Several factors including harvesting time of the aromatic plant, climatic and agronomic conditions, vegetative development of the plant, the plant part used, type of extraction used can be considered as responsible for fluctuations in their chemical compositions.
